# Supplementary material for: A multi-platform metabolomics approach identifies highly specific biomarkers of bacterial diversity in the vagina of pregnant and non-pregnant women
Source: Sci Rep. 2015 Sep 21;5:14174. doi: 10.1038/srep14174 (PMC4585667; doi:10.1038/srep14174)
Supplement: Supplementary Figures [file srep14174-s1.pdf]

A multi-platform metabolomics approach identifies highly specific biomarkers of bacterial diversity in the vagina of pregnant and non-pregnant women.

Amy McMillan (1,2), Stephen Rulisa (3), Mark Sumarah (4), Jean M. Macklaim (1,5), Justin Renaud (4), Jordan E. Bisanz (1,2), Gregory B. Gloor (5), and Gregor Reid (1,2,6)*

1.Canadian Centre for Human Microbiome and Probiotic Research, Lawson Health Research Institute, Western University, London, Ontario, Canada
2.Department of Microbiology and Immunology, Western University, London, Ontario, Canada
3.University of Rwanda, and University Teaching Hospital of Kigali, Kigali, Rwanda
4.Agriculture and Agri-food Canada, London, Ontario, Canada
5.Department of Biochemistry, Western University, London, Ontario, Canada
6.Department of Surgery, Western University, London, Ontario, Canada

*gregor@uwo.ca

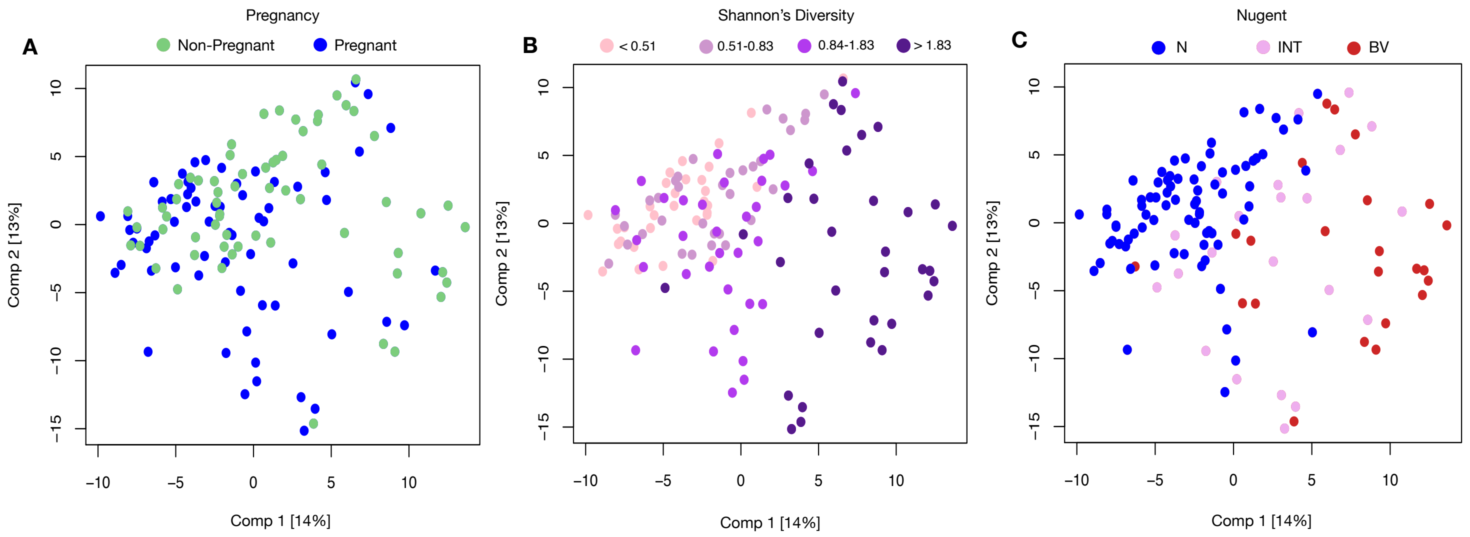

Supplementary Fig. S1. **Principal Component Analysis (PCA) scoreplots generated from 128 metabolites detected by GC-MS.** Each point represents a single sample from a single woman. Points are colored according to (**A**) pregnancy status, (**B**) the diversity of the microbiota measured using the Shannon index, or (**C**) Nugent score.

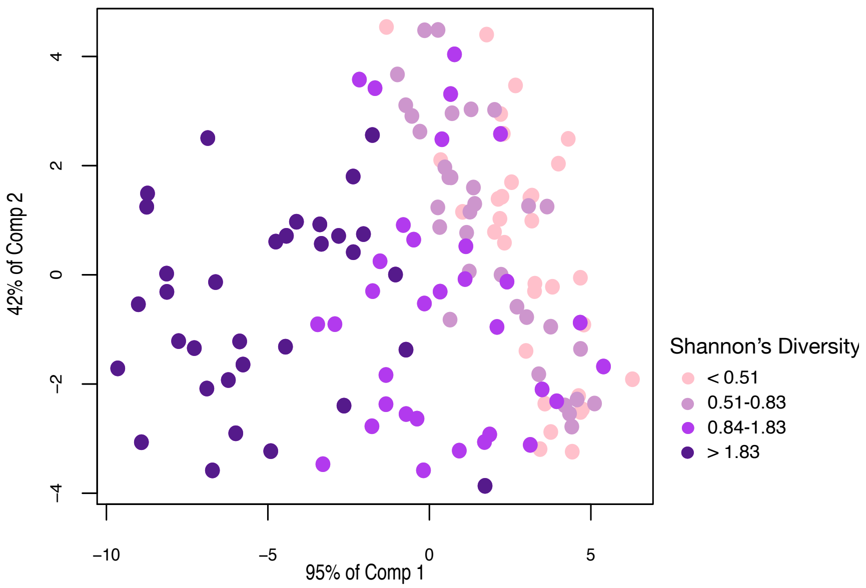

Supplementary Fig. S2. Combined cohort PLS regression scoreplot. PLS was built from 128 metabolites detected by GC-MS using bacterial diversity as a continuous latent variable. Each point represents a single woman (n=131). The position of points display similarities in the metabolome, with samples closest to one another being most similar. Circles are colored by diversity of the microbiota measured using the Shannon Index, where darker circles indicate higher diversity.

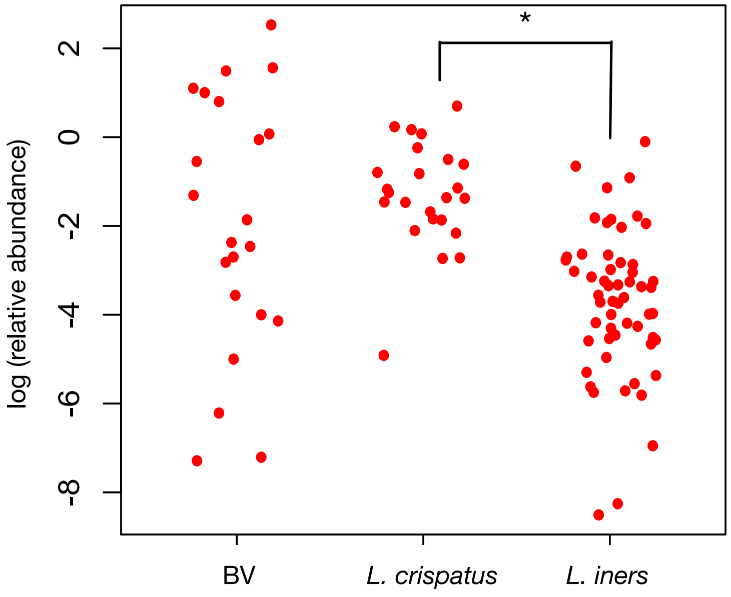

Supplementary Fig. S3. Relative abundance of succinate in women dominated by *L. crispatus, L. iners* or Nugent BV detected by GC-MS. (*) $p < 0.01$, unpaired t-test, Benjamini-Hochberg corrected.

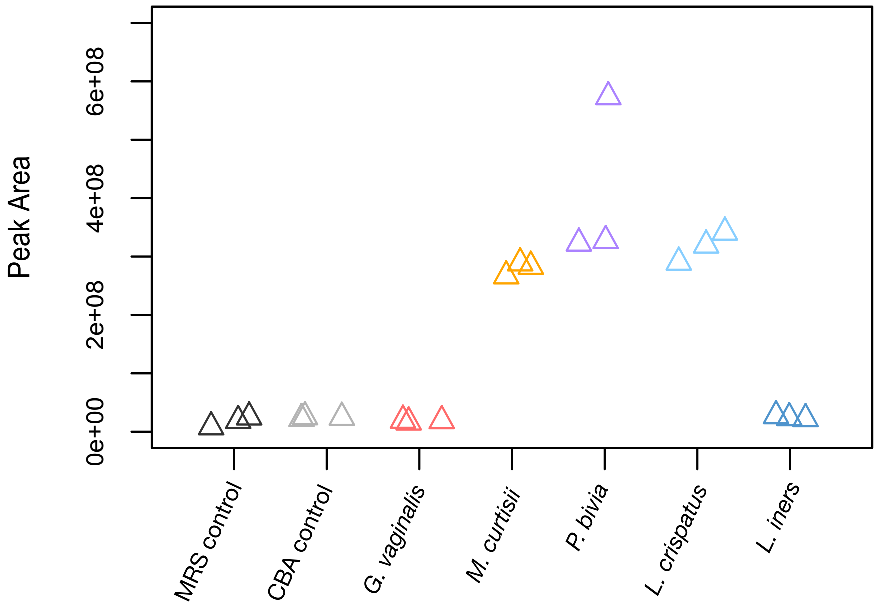

Supplementary Fig. S4. Succinate production by vaginal isolates. Bacteria were grown on agar plates and succinate detected by GC-MS. Three biological replicates are shown in technical duplicate. CBA: Columbia Blood Agar, MRS: de Man Rogosa Sharp Agar.
