# Supplementary material for: A multi-platform metabolomics approach identifies highly specific biomarkers of bacterial diversity in the vagina of pregnant and non-pregnant women
Source: Sci Rep. 2015 Sep 21;5:14174. doi: 10.1038/srep14174 (PMC4585667; doi:10.1038/srep14174)
Supplement: Supplementary Table S3 [file srep14174-s4.doc]

**Supplementary Table S3.** Percentage of variation in the metabolome that can be explained by a given variable. The percent variation explained by the x-axis (Component 1) of PLS regression plots are shown, where each variable was used as an independent continuous latent variable.

| **Variable** | **Pregnant**  **Comp1[%]** | **Non-Pregnant**  **Comp1[%]** |
| --- | --- | --- |
| Shannon's Diversity | 9.081732 | 10.668774 |
| Nugent | 6.659424 | 10.235316 |
| pH | 6.089376 | 9.010365 |
| *Lactobacillus* | 6.985202 | 10.048794 |
| *Gardnerella* | 4.233945 | 4.41815541 |
| *Prevotella* | 6.69468 | 7.938502 |
| *Atopobium* | 5.324604 | 6.107562 |
| *Dialister* | 8.610902 | 6.247007 |
| *Megasphaera* | 3.72020232 | 4.61373 |
| Sample ID | 5.29943535 | 6.480972 |
